# The thermodynamic profile and molecular interactions of a C(9)-cytisine derivative-binding acetylcholine-binding protein from *Aplysia californica*

**DOI:** 10.1107/S2053230X20001168

**Published:** 2020-02-03

**Authors:** Samuel Davis, Hugo Rego Campello, Timothy Gallagher, William N. Hunter

**Affiliations:** a Division of Biological Chemistry and Drug Discovery, School of Life Sciences, University of Dundee, Dow Street, Dundee DD1 5EH, Scotland; b School of Chemistry, University of Bristol, Bristol BS8 1TS, England

**Keywords:** acetylcholine-binding protein, crystal structure, cytisine, ligand-gated ion channel, nicotine

## Abstract

Cytisine, a natural product with high affinity for clinically relevant nicotinic acetylcholine receptors (nAChRs), is used as a smoking-cessation agent. The compound displays an excellent clinical profile and hence there is an interest in derivatives that may be further improved or find use in the treatment of other conditions. Here, the binding of a cytisine derivative modified by the addition of a 3-(hydroxypropyl) moiety (ligand **4**) to *Aplysia californica* acetylcholine-binding protein (*Ac*AChBP), a surrogate for nAChR orthosteric binding sites, was investigated. Isothermal titration calorimetry revealed that the favorable binding of cytisine and its derivative to *Ac*AChBP is driven by the enthalpic contribution, which dominates an unfavorable entropic component. Although ligand **4** had a less unfavorable entropic contribution compared with cytisine, the affinity for *Ac*AChBP was significantly diminished owing to the magnitude of the reduction in the enthalpic component. The high-resolution crystal structure of the *Ac*AChBP–**4** complex indicated close similarities in the protein–ligand interactions involving the parts of **4** common to cytisine. The point of difference, the 3-(hydroxypropyl) substituent, appears to influence the conformation of the Met133 side chain and helps to form an ordered solvent structure at the edge of the orthosteric binding site.

## Abstract 2

The binding of a C(9)-cytisine derivative carrying a 3-(hydroxypropyl) modification to acetylcholine-binding protein was characterized using isothermal titration calorimetry and crystallography.

## 1. Introduction   

**Figure 1:**
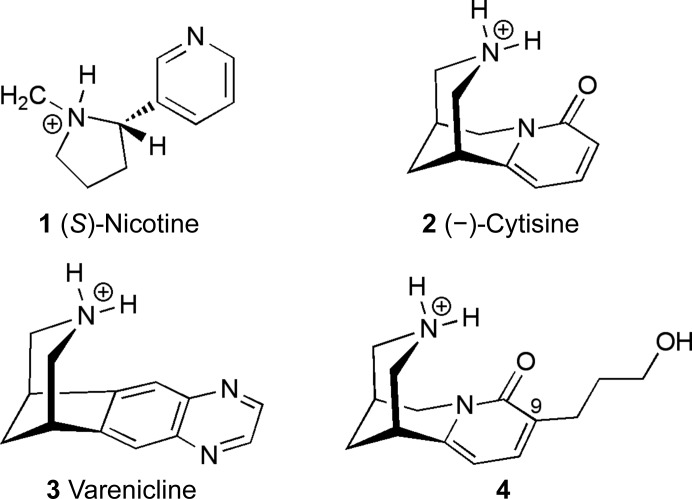
The chemical structures of nicotine {**1**; 3-[(2*S*)-1-methyl-2-pyrrolidinyl]pyridine}, cytisine [**2**; {(1*R*,5*S*)-1,2,3,4,5,6-hexahydro-1,5-methano-8*H*-pyrido[1,2a][1,5]diazocin-8-one}, varenicline {**3**; (1*R*,12*S*)-5,8,14-triazatetracyclo[10.3.1.0^2,11^0.0^4,9^]hexadeca-2(11),3,5,7,9-pentaene} and ligand **4** {(1*R*,5*S*)-9-(3-hydroxypropyl)-1,2,3,4,5,6-hexahydro-1,5-methano-8*H*-pyrido[1,2a][1,5]diazocin-8-one}.

Nicotine (**1**; Fig. 1 ▸) is the archetypical ligand of nicotinic acetylcholine receptors (nAChRs), a family of excitatory pentameric ligand-gated ion channels (pLGICs) that contribute to the function of the human peripheral and central nervous system. This family of receptors is being studied as they may represent therapeutic targets for Alzheimer’s disease, Parkinson’s disease and anti-nociception (Quik & Wonnacott, 2011 ▸; Umana *et al.*, 2013 ▸; Lombardo & Maskos, 2015 ▸). Nicotine is notorious as the agent responsible for the addictive effects of tobacco smoking, which worldwide is estimated to have caused six million deaths and 150 million disability-adjusted life years in 2015, and these numbers are increasing (GBD 2015 Tobacco Collaborators, 2017 ▸).

Cytisine (**2**; Fig. 1 ▸) is a natural product widely distributed in *Cytisus* and *Laburnum* species and is the active component in the smoking-cessation agent called Tabex, which is used widely in Central and Eastern Europe. Small-scale trials comparing cytisine with other smoking-cessation therapies have suggested that it has an excellent profile in terms of cessation rate, limited side effects and economical value, and this has raised interest in the potential use of cytisine in the wider global market (Hajek *et al.*, 2013 ▸; Walker *et al.*, 2014 ▸; West *et al.*, 2011 ▸). Cytisine, which is structurally related to nicotine, is a potent agonist at neuronal nAChRs but differs in displaying only partial agonist activity at α_4_β_2_ nAChRs, the high-affinity nicotine subtype. It is this combination of high affinity and partial agonism which has been identified as a hallmark of clinically successful smoking-cessation agents. Cytisine, however, also has significant full agonist activity at the heteromeric α_3_β_4_ and in particular the homomeric α_7_ nAChRs, which can induce off-target effects (Coe *et al.*, 2005 ▸; Rego Campello *et al.*, 2018 ▸). It is of interest therefore to understand how cytisine and its derivatives might interact with nAChR subtypes and to use this information to guide modifications that may improve its therapeutic profile. In one such approach cytisine provided the lead for varenicline (**3**; Fig. 1 ▸), a smoking-cessation agent marketed as Champix (Coe *et al.*, 2005 ▸). The C(9)-substituted ligand **4** (Fig. 1 ▸) is a derivative of cytisine carrying a 3-(hydroxypropyl) moiety on the pyridone ring. This offers an additional set of potential interactions involving the variable complementary β-subunit, and it is this region of the cytisine scaffold that has been associated with enhanced receptor-subtype discrimination (Rego Campello *et al.*, 2018 ▸). In this study, we sought to compare the binding parameters and structural interactions of cytisine and ligand **4** using acetylcholine-binding protein (AChBP). AChBP is a soluble, highly conserved homolog of the nAChR extracellular domain (ECD) in mollusks such as the Californian sea hare (*Aplysia californica*) and can therefore be used as a surrogate for crystallography and binding studies (see, for example, Shahsavar *et al.*, 2016 ▸; Dawson *et al.*, 2019 ▸; Smit *et al.*, 2003 ▸).

## 2. Materials and methods   

### 2.1. Protein production   

A recombinant source of *Ac*AChBP (UniProt ID Q8WSF8) with a C-terminal Tobacco etch virus cleavage site and His_6_ tag was produced in baculovirus-infected *Sf*9 insect cells using the Bac-to-Bac system (Thermo Fisher). Suspension High Five insect cells, cultured in Express Five medium plus 100 U ml^−1^ penicillin/streptomycin and 2 m*M* l-glutamine (Thermo Fisher), were used for protein production. 15 × 10^5^ cells per millilitre were infected with 5% of the baculovirus and incubated at 27°C in shaking flasks for 48 h before being harvested by centrifugation (1500*g* for 10 min at 12°C followed by 4000*g* for 10 min at 12°C). The *Ac*AChBP was secreted out to the medium and, using a Sartojet system with a 10 kDa cutoff Sartocon Slice filter (Sartorius), the medium was exchanged for buffer *A* (50 m*M* Tris–HCl, 250 m*M* NaCl pH 7.5) and the sample was concentrated. The protein solution was applied onto a 5 ml Ni^2+^ HisTrap column (GE Life Sciences) equilibrated in buffer *A* for immobilized metal-affinity chromatography. The column was washed with 15 column volumes of buffer *A* plus 7.5% buffer *B* (50 m*M* Tris–HCl, 250 m*M* NaCl, 800 m*M* imidazole pH 7.5) and the product was then eluted over 30 column volumes using a combination of a stepped and a linear gradient of buffer *B*. Fractions were analyzed by stain-free SDS–PAGE gels (Bio-Rad) and those containing the desired product were pooled, exchanged into buffer *A* and concentrated using 10 kDa centrifugal concentrators (Pall). Protein destined for use in ITC was dialyzed against buffer *A* using SnakeSkin 10 kDa dialysis tubing (Thermo Fisher) prior to concentration.

### 2.2. Isothermal titration calorimetry (ITC)   

ITC was carried out on a MicroCal PEAQ-ITC instrument (Malvern Panalytical). Ligands were dissolved in DMSO as 100 m*M* stocks and the concentration of DMSO was then matched in the titrant and cell solutions to minimize heat changes from buffer mismatch. Experiments utilized 12 × 3.0 µl or 15 × 2.5 µl injections, with the reference response of ligand titrated into buffer being subtracted. All experiments used a cell temperature of 25°C and the data were fitted with a one-site model using the manufacturer’s software.

### 2.3. Crystallization   

**Figure 2:**
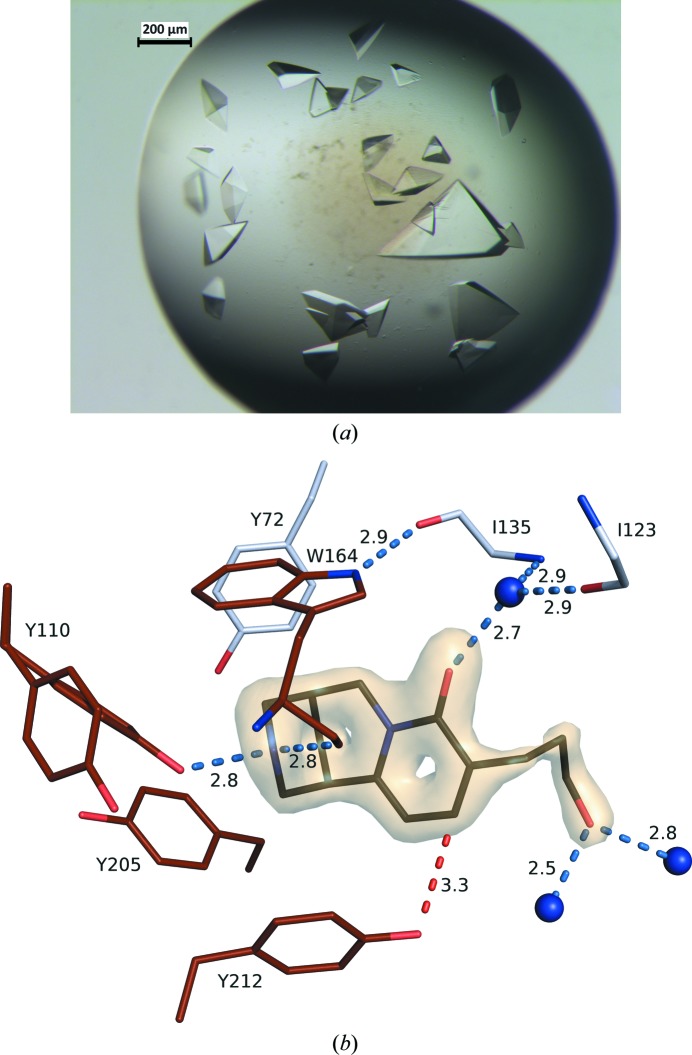
(*a*) Hanging drop containing crystals of the *Ac*AChBP–**4** complex. The largest crystal provided the data used for structure determination. (*b*) An OMIT difference density map for ligand **4** contoured at 6.5 standard deviations (associated with subunit *A*). C-atom positions of residues on the principal side of the binding site (subunit *A*) are shown in brown, those of residues on the complementary side (subunit *D*) are in gray and those for ligand **4** are in black. N and O atoms are colored blue and red, respectively. Three water molecules are depicted as blue spheres. Potential hydrogen bonds are identified with blue dashed lines and a C—H⋯O interaction is identified with a red dashed line. Two rotamers for Tyr110 are shown. Distances are in Å.

Initial crystallization trials were carried out by mixing 0.1 µl protein solution with 0.1 µl reservoir solution using MRC sitting-drop vapor-diffusion plates. A Mosquito crystallization robot (TPP) was used to prepare the drops, utilizing the JCSG-*plus* and Morpheus crystal screens (Molecular Dimensions), with protein solutions of 4 and 10 mg ml^−1^ *Ac*AChBP incubated with cytisine (3 m*M*) or ligand **4** (5 m*M*) for 1 h at room temperature. The synthesis of **4** will be described elsewhere, but is based on the use of enantiomerically pure N-Boc 9-bromocytisine as a substrate for a Pd(0)-mediated cross-coupling to an alkyl boronate ester (Rouden *et al.*, 2014 ▸). Seeking to increase the number of hits and improve the quality of the crystals, random matrix microseeding was implemented as described by Shaw Stewart *et al.* (2011 ▸). Seeded plates used a protein solution of 12.5 mg ml^−1^ plus 6 m*M* ligand **4** in 0.6 µl drops (0.3 µl protein solution plus 0.2 µl reservoir plus 0.1 µl seed-stock mixture). Subsequent optimization of the identified conditions used 24-well hanging-drop plates with 2 µl drops (all including 0.3 µl seed stock), screening the effect of drop ratios, precipitant concentration and additives (Table 1 ▸, Fig. 2 ▸ *a*). The crystal used for analysis grew in a reservoir consisting of 0.8 *M* NaH_2_PO_4_, 0.8 *M* KH_2_PO_4_, 10% glycerol, 0.1 *M* HEPES pH 7.0, with the drop consisting of 1.5 µl protein solution and 0.2 µl reservoir solution.

### 2.4. Crystallographic analyses   

Crystals were harvested with a nylon loop, cryoprotected in 20% glycerol made up in the reservoir solution and then snap-frozen in liquid N_2_. Diffraction data were recorded on beamline I03 at Diamond Light Source (DLS; Table 2 ▸), indexed and integrated with *DIALS* (Winter *et al.*, 2018 ▸) and scaled and merged in *AIMLESS* (Evans & Murshudov, 2013 ▸). The structure was solved by molecular replacement with *Phaser* (McCoy *et al.*, 2007 ▸) using the 2.20 Å resolution structure of *Ac*AChBP in complex with an epibatidine derivative as the search model (PDB entry 6qkk; S. Davis, R. V. Bueno, A. Dawson & W. N. Hunter, unpublished work). *REFMAC* v.5.8.0257 (Murshudov *et al.*, 2011 ▸) was used for multiple rounds of automated restrained refinement, with manual refinement and model building in *Coot* (Emsley *et al.*, 2010 ▸). *MolProbity* (Chen *et al.*, 2010 ▸) was used for Ramachandran analysis. Restraints and models for ligand **4** were generated using the *Grade* server (Global Phasing; http://grade.globalphasing.org/cgi-bin/grade/server.cgi) and graphics were rendered using the *PyMOL* molecular-graphics system (Schrödinger). Two pentamers were present in the asymmetric unit and the ligand **4** was unambiguously placed in every binding site on the basis of electron and difference density (see, for example, Fig. 2 ▸ *b*). The protein is glycosylated and *N*-acetyl-d-glucosamine was modeled onto Asn91 in every subunit. Strict local noncrystallographic symmetry restraints were initially applied, but were then relaxed during the course of refinement. It became evident that multiple conformations were visible for certain residues and these were manually assigned, with different occupancies being tested until the difference density maps suggested appropriate modeling. During the placement of water molecules it was apparent that ordered ions and other ligands were also present, and these were assigned as Cl^−^, K^+^, glycerol or phosphate. Crystallo­graphic statistics are given in Table 3 ▸.

## 3. Results and discussion   

### 3.1. Thermodynamic parameters   

**Figure 3:**
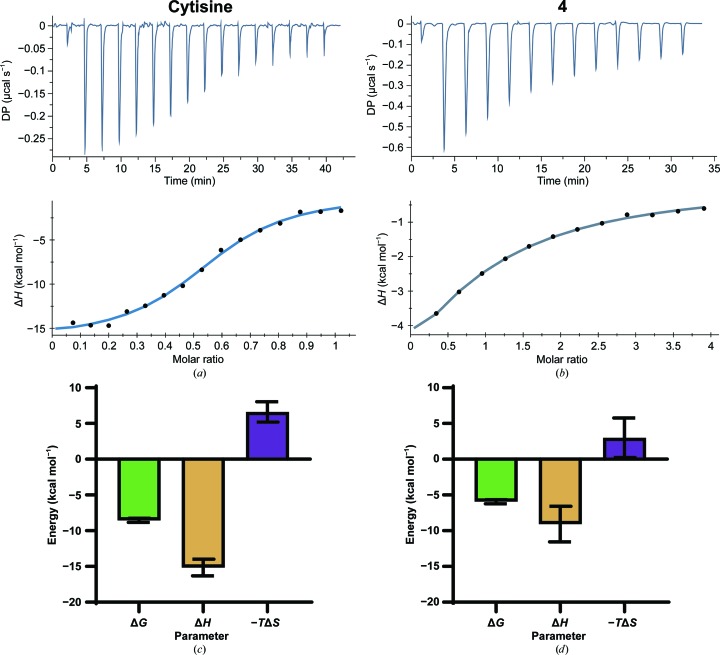
Raw and injection heat-normalized thermodynamic traces for the binding of (*a*) cytisine and (*b*) ligand **4** to *Ac*AChBP and (*c*, *d*) the corresponding signature plots. Error bars represent the standard errors of the mean.

The ITC measurements for cytisine (**2**) binding to *Ac*AChBP (Fig. 3 ▸, Table 4 ▸) are similar to those previously published (Rucktooa *et al.*, 2012 ▸). For example, the values of the thermodynamic dissociation constant *K*_d_ are 0.6 (±0.3) and 1.6 µ*M*, respectively, and a similar thermodynamic profile is obtained that indicates that the binding is dominated by a favorable enthalpic contribution. In the present work Δ*H* was measured as −15.2 (±1.2) kcal mol^−1^, whilst in the previous study the value was −13.3 kcal mol^−1^. The small differences observed are likely to be owing to the use of different buffers (see, for example, Celie *et al.*, 2004 ▸). The affinity of ligand **4** is significantly reduced compared with that of **2**, with *K*_d_ values of 53.3 (±19.9) and 0.6 (±0.3) µ*M*, respectively. For both **2** and **4** there is an unfavorable entropic contribution of 6.6 (±1.4) and 3.0 (±2.8) kcal mol^−1^, respectively, but binding is driven by gains in enthalpy, as discussed above. For **4**, although the entropic term is less unfavorable, the enthalpic contribution is reduced and therefore the affinity is lowered. This is consistent with the general reduction in binding affinity of nAChRs for cytisine derivatives possessing pyridone-ring modifications (Rego Campello *et al.*, 2018 ▸).

Ligand **4** displays a stoichiometry of approximately 4.5:1 for interaction with the pentameric *Ac*AChBP (Table 3 ▸), which is close to the expected 5:1 ratio for a ligand that fully occupies the binding site in a straightforward association. We note, however, that for **2** the stoichiometry is about 2.8:1. Our data are strikingly consistent with the previous study of *Ac*AChBP interacting with **2** (see Fig. 3B in Rucktooa *et al.*, 2012 ▸). It has previously been noted, again using ITC (Celie *et al.*, 2004 ▸), that carbamylcholine binds the pentameric *Ac*AChBP at a molar ratio of 2.5:1. The agonist carbamylcholine displays a similar affinity for *Ac*AChBP as **2**, 7.6 ± 0.4 µ*M*, and the reason for the low molar ratio is unclear. The finger of suspicion would point towards experimental issues such as the presence of impurities, uncertainty in the concentrations of the ligand and/or protein, the degradation of reagents or precipitation/aggregation (a particular problem at high concentrations). Alternatively, cooperativity or allosteric transitions can complicate the analyses. We have not uncovered any proof for such behavior in *Ac*AChBP in this or previous studies (Dawson *et al.*, 2019 ▸; Jones *et al.*, 2020 ▸) and are unable to shed further light on these observations relating to carbamylcholine or **2**.

### 3.2. A seeding route for suitable crystals   

Attempts to directly co-crystallize ligand **4** with *Ac*AChBP were unsuccessful. Therefore, *Ac*AChBP was co-crystallized with 3 m*M* cytisine for random matrix microseeding. Small crystals grew in a drop consisting of 5 mg ml^−1^ protein and 0.4 *M* NaH_2_PO_4_, 0.4 *M* KH_2_PO_4_, 0.05 *M* HEPES pH 7.5. These crystals were crushed and used to seed drops consisting of 6.25 mg ml^−1^ *Ac*AChBP plus 3 m*M* ligand **4** and 3% DMSO. This generated crystals in the same reservoir conditions as the originating seed stock. Subsequent optimization in 24-well hanging-drop plates led to crystals with maximal dimensions of ∼400 × 400 × 300 µm in 0.8 *M* NaH_2_PO_4_, 0.8 *M* KH_2_PO_4_, 10% glycerol, 0.1 *M* HEPES pH 7.0 (Fig. 3 ▸). The drop consisted of 1.5 µl protein solution (12.5 mg ml^−1^ *Ac*AChBP, 6 m*M* ligand **4**, buffer *A*), 0.2 µl reservoir solution (0.8 *M* NaH_2_PO_4_, 0.8 *M* KH_2_PO_4_, 10% glycerol, 0.1 *M* HEPES pH 7.0) and 0.3 µl seed stock (Fig. 2 ▸ *a*). High-quality diffraction data were measured using synchrotron radiation and the structure was solved and refined to our satisfaction.

### 3.3. Structure of the *Ac*AChBP–**4** complex   

The monoclinic crystal has two pentameric assemblies in the asymmetric unit. A high degree of noncrystallographic symmetry is evident even though NCS restraints were released in the refinement calculations. Electron density for the ligand is well defined in all ten binding sites and they refined with average *B* factors less than or close to the values noted for their associated subunits (Table 3 ▸). The position of the ligand and interactions formed with the protein are highly conserved in each binding site and it is only necessary to detail one.

There are two crystal structures with cytisine in the Protein Data Bank (PDB) that are relevant to our study; a low-resolution (2.9 Å) complex with *Ac*AChBP (PDB entry 4bqt; Rucktooa *et al.*, 2012 ▸) and a 2.0 Å resolution structure (PDB entry 5syo; J. Bobango, J. Wu, I. T. Talley & T. T. Talley, unpublished work) in which loop C has been engineered to incorporate amino acids from the human α_3_ nAChR. Also of note is the 2.0 Å resolution complex of varencline with *Capitella telata* AChBP (PDB entry 4afg; Billen *et al.*, 2012 ▸).

**Figure 4:**
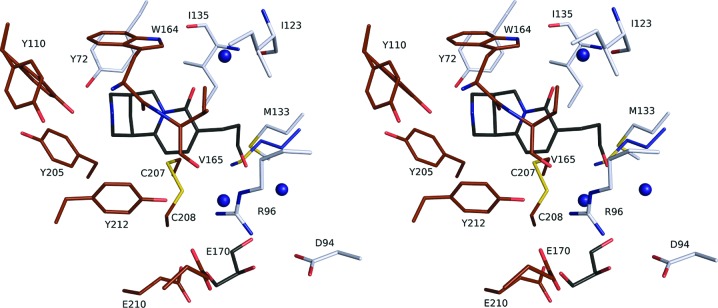
Stereoview of ligand **4** in a representative *Ac*AChBP binding site. In this case, the site is formed between subunit *A* (principal side) and subunit *D* (complementary side). A similar color scheme to Fig. 2 ▸(*b*) is used with the following additions: S-atom positions are shown in yellow, glycerol C atoms are shown in black and the side chain of a Met133 rotamer from a complex with cytisine (PDB entry 5syo) directed into the binding site is shown with blue C atoms.

The interactions of ligand **4** in the *Ac*AChBP binding site are similar to those of cytisine (**2**) and other nAChR ligands, such as varenicline (**3**; Fig. 4 ▸; Billen *et al.*, 2012 ▸; Rucktooa *et al.*, 2012 ▸; Dawson *et al.*, 2019 ▸). The Tyr110 OH group and the carbonyl backbone of Trp164 accept hydrogen bonds from the secondary amine of the ligand, indicating the presence of a protonated group. The Tyr110 OH group also donates a hydrogen bond to the carbonyl group of Ser163. The alignment and distances of between 4.6 and 4.2 Å of the aromatic groups on Trp164 and Tyr212, respectively, from the nitrogen suggest that cation–π interactions contribute to binding. Interestingly, dual rotamers of Tyr110 were modeled in seven out of ten subunits. The main rotamer, with full occupancy in three subunits and an occupancy of between 60% and 80% in the remainder, is that which interacts as described above and is shown in Fig. 4 ▸. In two cases the second rotamer is only slightly different and still participates in similar interactions. The rest, in five subunits, adopt a conformer with the OH group too far away for hydrogen bonding to the ligand. This rotamer, with occupancies of between 20% and 40%, is that observed in the apo-form binding site; for example in the three vacant binding sites of PDB entry 5syo. The orthosteric binding site of the pLGIC family possesses a degree of plasticity to accommodate ligands of differing properties, and this is noted in particular for those residues that constitute part of loop C (see, for example, Dawson *et al.*, 2019 ▸). That we also note the presence of different rotamers for the side chain of Tyr110 suggests that here also deep in the binding site there may be a degree of conformational freedom for part of the aromatic cage.

Tyr205 and Tyr212 participate in van der Waals interactions with the pyridone ring and with a potential C—H⋯O interaction involving the Tyr212 OH group. The pyridone C=O is about 3.5 Å from Trp164 NE1 but the geometry is not optimal for hydrogen bonding. The ligand carbonyl group accepts a hydrogen bond, with a distance of 2.7 Å, donated from a water molecule that then bridges to Ile135 (via the carbonyl) and Ile123 (via the amide) on the complementary side of the binding site. This hydration point is highly conserved in *Ac*AChBP and nAChR structures, and appears to partially mediate ligand affinity and possibly the mode of action (Billen *et al.*, 2012 ▸; Zhang *et al.*, 2012 ▸). The only available *Ac*AChBP–cytisine structure (PDB entry 4bqt; Rucktooa *et al.*, 2012 ▸) is of low resolution (2.9 Å) and does not show any water molecules in the binding site. However, the higher resolution engineered *Ac*AChBP–cytisine structure (PDB entry 5syo) with loop C altered to mimic the human α_3_ nAChR structure shows a water molecule in the same position hydrogen-bonded to the cytisine C=O and the complementary side residues as per the ligand **4** complex structure.

Ligand **4** is primarily hydrophobic, and van der Waals interactions, which are a major determinant of binding, between the ring systems and the protein involve Trp164, Tyr72, Tyr205, Tyr212, Ile135 and Val165, and the disulfide between Cys207 and Cys208. The 3-hydroxylpropyl substituent interacts with Val125, Ile135, the Cys207–Cys208 disulfide, the side chain of Met133 and the main chain of Phe134. The hydroxyl residue on **4** is directed out of the binding site, forming hydrogen bonds to two well ordered water molecules, which in turn interact with other waters and glycerol. A glycerol is observed in all ten orthosteric binding sites of the asymmetric unit and occupies a highly polar pocket created by the side chains of Asp94, Arg96, Glu170 and Glu210.

The possibility of influencing affinity by virtue of exploiting waters that are able to form hydrogen-bonding networks to link the ligand to the protein was a consideration in the design of **4**. However, although the presence of such well ordered waters may contribute to the formation of a stable complex, the ITC data indicate that the 3-propylalcohol substituent leads to a reduced ligand affinity. The program *Torsion­Analyzer* (Schärfer *et al.*, 2013 ▸) was used to investigate the conformation of the 3-propylalcohol substituent and to compare it with structures in the Cambridge Crystallographic Database. The two torsion angles relevant to the conformation of the aliphatic C atoms display values close to −60° for all ten ligands in the asymmetric unit. This matches the well characterized minima expected for a staggered conformation; moreover, this represents the most prevalent conformation in the database. This suggests that the ligand has adopted a preferred low-energy conformation. Nevertheless, the inclusion of the substituent has reduced the affinity for *Ac*AChBP. An overlay of the complexes with cytisine and **4** identifies structural perturbation involving Met133. In the cytisine complexes the side chain of this residue displays a rotamer that is directed into the binding site and is able to form van der Waals inter­actions with the ligand. In the *Ac*AChBP–**4** complex we note that the 3-hydroxylpropyl substituent appears to force the Met133 side chain to adopt different rotamers and thus avoid steric clash. This may influence the thermodynamic profile of ligand **4** compared with the cytisine by virtue of introducing a degree of strain into the protein structure coupled with a change to the hydration structure within the binding site. Of note is that in a human α_4_β_2_ nAChR orthosteric site the residue that corresponds to Met133 in *Ac*AChBP aligns with Gln150 or Phe144 in the α_4_ and β_2_ subtypes, respectively. Modification of the cytisine framework to enhance activity might have to consider the likelihood of unfavorable interactions with a sizable and flexible side chain in this part of the binding site.

## 4. Conclusions   

We have characterized the binding of cytisine and ligand **4**, a novel 9-substitued cytisine variant, to *Ac*AChBP using ITC and reported the high-resolution crystal structure of the *Ac*AChBP–**4** complex. Ligand **4** carries a 9-(3-hydroxypropyl) modification and this reduces its affinity for *Ac*AChBP. Structural comparisons indicate that the molecular inter­actions involving the identical components of cytisine and ligand **4** are conserved. There is only a minor perturbation of the protein structure at the side chain of Met133, where a steric clash is likely to contribute to the reduced affinity of **4** compared with the parent compound. The complex structure suggests that recovery of affinity may be possible with substituents that interact with a polar patch at the periphery of the binding site.

## Supplementary Material

PDB reference: acetylcholine-binding protein–ligand complex, 6t9r

## Figures and Tables

**Table 1:** Crystallization of the *Ac*AChBP–**4** complex

Method	Vapor diffusion
Plate type	Hanging drop (Hampton Research)
Temperature (K)	295
Protein concentration (mg ml^−1^)	12.5
Buffer composition of protein solution	50 m*M* Tris–HCl, 250 m*M* NaCl pH 7.5 + 6 m*M* ligand **4**
Composition of reservoir solution	0.8 *M* NaH_2_PO_4_, 0.8 *M* KH_2_PO_4_, 10% glycerol, 0.1 *M* HEPES pH 7.0
Volume and ratio of drop	1.5 µl protein solution:0.2 µl reservoir solution:0.3 µl seed stock
Volume of reservoir (µl)	650

**Table 2:** Diffraction data-collection parameters

Diffraction source	Beamline I03, DLS
Wavelength (Å)	0.976
Temperature (K)	100
Detector	EIGER2 XE 16M
Crystal-to-detector distance (mm)	288.18
Rotation range per image (°)	0.1
Total rotation range (°)	180

**Table 3:** Crystallographic statistics for the *Ac*AChBP–**4** complex Values in parentheses are for the highest resolution shell.

Data statistics	
Unit-cell parameters (Å, °)	*a* = 209.5, *b* = 132.9, *c* = 131.1, α = 90.0, β = 102.5, γ = 90.0
Space group	*C*2
Wavelength (Å)	0.976
Subunits per asymmetric unit	10
Resolution range (Å)	127.97–1.72 (1.75–1.72)
Total No. of reflections	1225936 (25553)
Unique reflections	353432 (12162)
Multiplicity	3.5 (2.1)
*R*_merge_ †	0.07 (0.82)
*R*_p.i.m._	0.064 (0.794)
Wilson *B* factor (Å^2^)	25.07
Completeness (%)	95.5 (66.6)‡
〈*I*/σ(*I*)〉	7.6 (0.6)§
CC_1/2_ ¶	0.993 (0.435)
Refinement	
Resolution range (Å)	90.12–1.72
*R*_work_/*R*_free_ †† (%)	16.2/19.2
No. of reflections for *R*_work_/*R*_free_	335977/17388
No. of protein residues	2058
No. of NAG molecules	10
No. of molecules of **4**	10
No. of phosphate molecules	10
No. of glycerol molecules	40
No. of water molecules	2899
No. of chloride ions	10
No. of potassium ions	10
R.m.s.d.s	
Bond lengths (Å)	0.013
Angles (°)	1.84
Ramachandran plot	
Residues in favored regions (%)	98.77
Residues in allowed regions (%)	1.23
Mean *B* factors (Å^2^)	
Protein atoms (subunit *A*–*J*)	30.1/29.2/29.7/28.4/30.6/29.8/31.5/31.8/33.4/34.5
NAG molecules (subunit *A*–*J*)	93.7/90.2/108.6/87.6/99.5/97.1/104.2/103.6/100.2/92.6
Water molecules	46.0
Ligand **4** (subunit *A*–*J*)	28.1/27.9/23.5/22.2/27.9/29.9/23.7/32.4/33.3/31.5
Phosphate ions (subunit *A*–*J*)	58.1/54.2/57.4/63.8/49.2/49.5/56.4/71.9/52.6/58.0
Glycerol molecules	59.9
Chloride ions (subunit *A*–*J*)	33.7/34.6/32.0/32.9/33.5/34.0/37.9/35.2/35.9/37.8
Potassium ions (subunit *A*–*J*)	37.3/37.4/38.6/40.0/39.5/40.5/41.3/40.5/43.2/44.2
PDB code	6t9r

† *R*_merge_ = 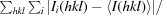 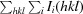, where *I_i_*(*hkl*) is the intensity of the *i*th measurement of reflection *hkl* and 〈*I*(*hkl*)〉 is the mean value of *I_i_*(*hkl*) for all *i* measurements.

‡ Completeness was <70% in the highest resolution shell owing to the use of a square detector for data collection.

§ 〈*I*/σ(*I*)〉 = 2.0 at 1.90 Å resolution.

¶ Pearson correlation coefficient.

†† *R*_work_ = 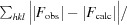 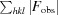, where *F*_obs_ is the observed structure-factor amplitude and *F*_calc_ is the structure-factor amplitude calculated from the model. *R*_free_ is the same as *R*_work_ except that it was calculated using a subset (5%) of data that were excluded from refinement calculations.

**Table 4:** Thermodynamic parameters Values in parentheses indicate the standard errors of the mean.

Ligand	Ligand concentration (µ*M*)	*Ac*AChBP concentration (µ*M*)	No. of sites	*K*_d_ (µ*M*)	Δ*G* (kcal mol^−1^)	Δ*H* (kcal mol^−1^)	−*T*Δ*S* (kcal mol^−1^)
Cytisine (**2**) (*n* = 2)	100	20	0.6 (0.01)	0.6 (0.3)	−8.6 (0.3)	−15.2 (1.2)	6.6 (1.4)
**4** (*n* = 3)	1000	50	0.9 (0.10)	53.3 (19.9)	−6.0 (0.3)	−9.1 (2.5)	3.0 (2.8)
